# Suicidality and Illness Course Worsening in a Male Patient with Bipolar Disorder during Tamoxifen Treatment for ER+/HER2+ Breast Cancer

**DOI:** 10.1155/2021/5547649

**Published:** 2021-03-24

**Authors:** Claudia Carmassi, Francesco Pardini, Valerio Dell'Oste, Annalisa Cordone, Virginia Pedrinelli, Marly Simoncini, Liliana Dell'Osso

**Affiliations:** ^1^Department of Clinical and Experimental Medicine, University of Pisa, Pisa, Italy; ^2^Department of Biotechnology, Chemistry and Pharmacy, University of Siena, Siena, Italy

## Abstract

**Purpose:**

Tamoxifen is a selective estrogenic receptor modulator (SERM) drug. In addition to its common use in breast cancer ER+, Tamoxifen has been object of growing interest in psychiatry as antimanic drug. At the same time, clinical concerns about Tamoxifen's depressogenic effect have been repeatedly raised even without reaching univocal conclusions. We discuss the case of a 45-year-old-male with a diagnosis of Bipolar Disorder type II, treated with Tamoxifen as relapse prevention treatment after surgery for a ER+/HER2+ breast cancer. The patient required two psychiatric admissions in a few-month time span since he showed a progressive worsening of both depressive and anxiety symptoms, with the onset of delusional ideas of hopelessness and failure up to suicidal thoughts. The clinical picture showed poor response to treatment trials based on various associations of mood-stabilising, antidepressants, and antipsychotic drugs. During the second hospitalization, after a multidisciplinary evaluation, the oncologists agreed on Tamoxifen discontinuation upon the severity of the psychiatric condition. The patient underwent a close oncological and psychiatric follow-up during the following 12 months.

**Methods:**

Psychiatric assessments included the Montgomery-Asberg Depression Rating Scale (MADRS), the Hamilton Depression Scale (HAM-D), the Columbia Suicide Severity Rating Scale (C-SSRS), and the Quality of Life Enjoyment and Satisfaction Questionnaire Short Form (Q-LES-Q-SF). All questionnaires were administered at the time of the second hospitalization and in a one-year follow-up.

**Results:**

Suicidal ideation fully remitted and depressive symptoms markedly and rapidly improved in the aftermath of Tamoxifen discontinuation. The symptomatological improvement remained stable across one-year follow-up.

**Conclusions:**

Male patients with a mood disorder history constitute a high-risk group as to Tamoxifen psychiatric side effects. The onset or worsening of depressive symptoms or suicidality should be carefully addressed and promptly treated, and clinicians should be encouraged to consider the possibility of discontinue or reduce Tamoxifen therapy after a multidisciplinary evaluation.

## 1. Introduction

Tamoxifen is a Selective Estrogen Receptor Modulator (SERM), a synthetic nonsteroidal drug characterized by a tissue specific pattern of action (antiestrogen in mammalian and blood tissue, estrogen-like in endometrial tissue, bones, and liver) [1]. The main therapeutic indications of Tamoxifen are the treatment and the chemoprevention of estrogenic receptor positive (ER+) breast cancer; consistently, it has been extensively prescribed in oncological setting since 1973 usually as long-term treatment [2].

Preclinical studies suggest that at the brain level, Tamoxifen may act as a partial agonist, with a prevalence of antagonist or agonist effect depending on the preexisting hormonal status [3, 4]. In this regard, it is worth mentioning that the antiestrogenic effect of Tamoxifen may reduce the postsynaptic response to serotonin and brain serotonin- and norepinephrine-related activity and antagonise serotonin and acetylcholine effects [3, 5, 6]. Indeed, from a clinical point of view, the prominent role of steroidal sexual hormones in affecting mood regulation and cognitive functioning is widely acknowledged [7]. The association between estrogen levels' fluctuations during lifespan, such as postpartum or perimenopausal, and vulnerability to depression or mood disorder has been repeatedly reported [8–10], while estrogenic treatments have been linked to hyperactivity or manic symptoms onset [11–14].

Clinical concerns about the risk of developing depressive symptoms or full-blown depressive episodes during Tamoxifen assumption have been repeatedly raised [15–17]. While some large cohort studies exploring this issue have yielded to conflicting results [17–22], literature abounds with case-report studies describing the onset or the exacerbation of depressive-type mood alterations while undergoing Tamoxifen therapy [23–29]. Some points emerging from these studies deserve particular attention. First, case-reports suggest that patients with underlying psychiatric vulnerability or a clinical history of mood disorder may be more vulnerable to the development of depression as Tamoxifen's side effect, and the clinical phenotype described in these studies is sometimes of remarkable severity, with a poor response to antidepressants trials [26, 27, 29] and a significant presence of suicidal thoughts [23, 25, 26] and behaviours [27]. In addition, while male patients undergoing a Tamoxifen therapy appear to be more likely to develop side effects, including psychiatric ones [30, 31], they are usually underrepresented in the studies addressing this issue.

More recently, the possibility of using Tamoxifen as novel antimanic drug has gained increasing interest [32]: this depends on the fact that Tamoxifen is a Protein Kinase C (PKC) inhibitor, which can cross brain-blood barrier and is highly tolerable even at high dose [33–35]. The clinical investigations unanimously indicated that Tamoxifen exerts a prompt and effective antimanic action [33, 36] and, accordingly, some guidelines [35] included Tamoxifen among acute mania therapeutic options [35]. However, it should be stressed that the long-term effect of Tamoxifen on Bipolar Disorder (BD) course is yet to be clarified, since the aforementioned studies cover a short observation period [33, 36].

The described scenario is complex and calls for further studies aiming to better clarify the effects of Tamoxifen on mood, especially considering on the one hand its wide use in oncological settings [37, 38] on patients who are highly heterogeneous as to psychiatric vulnerability, and on the other hand, its ever-growing use in psychiatry, as new antimanic agent [33, 36].

We report the description of the clinical case of a 45-year-old male with previous diagnosis of BD type II (BD-II) whose psychopathological conditions worsened during treatment with Tamoxifen for a breast cancer. Our aim is to emphasize the potential role of Tamoxifen in triggering depressive symptoms up to suicidal thoughts in a patient with BD.

## 2. Case Description

### 2.1. Clinical Details

The patient (XY) is a 45-year-old accountant, married with three children. He reported a family history of mood and anxiety disorders and had been previously diagnosed with DB-II in comorbidity with Panic Disorder (PD). While on Tamoxifen therapy for breast cancer, XY required two subsequent admissions to the Inpatient Unit of the University of Pisa Psychiatric Clinic over a five-month period, due to the progressive worsening of a major depressive episode associated with suicidal thoughts.

XY was treated for the onset of a PD with mild depressive symptoms at the age of 30. These symptoms determined XY's first contact with a psychiatrist who introduced the antidepressant Paroxetine, with full recovery within a few months and complete remission without any treatment for almost 10 years. During this time span, XY experienced at least two episodes of mood elevation of hypomanic intensity, characterized by increased energy level and working activities, and reduced need for sleep (about 4 hour at night) and more pressure in talking. These episodes showed duration of about 2 weeks and did not come to medical attention since the patient had no insight about their pathological nature, and adequate working and social functioning were conserved despite an increased irritability was noted by relatives and friends. Indeed, they were identified and reported only at a subsequent time, during anamnestic collection at the first hospital admission. In 2014, in relation to persistent familial and working stressors, XY experienced a progressive increase of anxiety associated to panic attacks, ruminative thinking about his problems, and low mood with irritability and insomnia. For this reason, he was prescribed the antidepressant Duloxetine, which did not produce any improvement and exacerbated anxiety, irritability, and internal tension. Duloxetine was thus discontinued, and XY switched to a combined therapy with mood stabilizer (Divalproex), antidepressant (Paroxetine), and benzodiazepines with a rapid clinical response and later reduction of the dosages up to a maintenance treatment. About two years later, XY was diagnosed with pT1bN0 G3 ER+/HER+ breast cancer: he underwent surgery and one and a half years adjuvant chemotherapic treatment. During this period, XY showed mild periodical exacerbations of depressive symptoms that remitted with minor changes to the ongoing therapy. Tamoxifen therapy was subsequently introduced (daily dose: 20 mg). After about 10 months from the introduction of Tamoxifen, XY showed a progressive psychopathological worsening with increasing anxiety, lower mood, and reduced energy, so that the psychiatrist tried different antidepressants trials (Escitalopram, Trimipramine, Fluoxetine) along with Divalproex, with a partial response. In January 2019, a further symptomatological exacerbation, with increased tension, low mood with lability, hopelessness, ruminative and obsessive thinking about physical, familiar, and working problems with concurrent worsening of suicidal ideation required the first admission to Psychiatric Clinic of University of Pisa. XY was clinically diagnosticated with BD-II comorbid with PD, and afterwards, he was discharged with Divalproex, Sertraline, and Olanzapine, showing partial clinical improvement; meanwhile, he continued undergoing a Tamoxifen therapy. In the following months, despite several treatment changes were undertaken, XY showed a gradual worsening of symptoms up to an aborted suicidal attempt by defenestration. The subsequent introduction of Lithium in addition to Divalproex, Citalopram, Quetiapine, and Delorazepam did not lead to any significant improvement, and suicidal ideation kept worsening. Therefore, approximately 5 months after the previous discharge, the patient needed hospital readmission. The ongoing therapy at the second admission was Divalproex 300 mg/day, Lithium Sulphate 41.5 mg/day, Citalopram 20 mg/day, and Quetiapine 75 mg/day. During the second hospitalization, he was assessed with the following instruments: the Structured Clinical Interview for DSM-5 Disorders (SCID-5) [39], a diagnostic instrument used by clinicians to make psychiatric diagnoses through a semistructured interviewing process, the Montgomery-Asberg Depression Rating Scale (MADRS) [40] and the Hamilton Depression Scale (HAM-D) [41], to evaluate depressive symptoms severity, the Columbia Suicide Severity Rating Scale (C-SSRS) [42], to assess suicidality, and the Quality of Life Enjoyment and Satisfaction Questionnaire Short Form (Q-LES-Q-SF) [43]. The clinical diagnosis of BD-II, comorbid with PD, was confirmed by means of the SCID-5 due to the lifetime presence of at least one full-blown depressive episode and of at least a hypomanic episode, described by the patient although never clinically observed. At the hospitalization, the C-SSRS showed the presence of suicidal ideation (intensity of 14 points) with lifetime, yet not current, suicidal behavior. During this hospitalization, XY was treated with mood stabilizers (Divalproex 500 mg and Lithium Sulfate 83 mg), antidepressant (Citalopram 20 mg, then Sertraline 125 mg), and antipsychotics (Quetiapine 125 mg, then Olanzapine 10 mg) with only moderate improvement of the symptoms. We then contacted the oncologist in order to consider the possibility of discontinuation of treatment with Tamoxifen. The oncologist agreed on the interruption of Tamoxifen treatment, as about 18 months had been already occurred since its first assumption. After the discharge, we started a close follow-up aimed at observing XY's clinical evolution and at repeating the psychometric assessments, without making any further therapeutic changes. At the following evaluations until today, XY has shown a global clinical improvement, with progressive reduction of depressive and anxious symptoms, reduction of ruminative concern about familiar and working problems, increasing energy level, complete absence of delusional ideation, and fully recover of his work capacity. No suicidal thoughts or plan have emerged so far. The scores reported in the subsequent assessments confirmed the clinical improvement (see Table 1), showing a progressive reduction of depressive symptoms and a global improvement in quality of life, mainly after Tamoxifen's suspension. To date, after 20 months from Tamoxifen interruption, Olanzapine and Divalproex have been discontinued and XY is on a Lithium and Paroxetine maintenance therapy. At the same time, the patient had a good oncological follow-up despite Tamoxifen interruption.

### 2.2. Assessment Instruments

The patient gave written informed consent to the processing of health data for research purposes and fulfilled the following scales.

The MADRS [40] is based on clinical interview with the patient; the interviewer is required to use clinical judgment to determine whether the rating lies on the defined scale steps (0, 2, 4, and 6 points) or between them (1, 3, and 5 points, denoted as “Worsening symptoms”). This scale consists of 10 items: apparent sadness, reported sadness, inner tension, reduced sleep, reduced appetite, concentration difficulty, lassitude, inability to feel, pessimistic thoughts, and suicidal thoughts. The total score is compared to the corresponding descriptor: 0-6 = normal; 7-19 = mild depression; 20-34 = moderate depression; 34-60 = severe depression.

The HAM-D [41] is a self-report measure of depressive symptoms that lists 21 items, the scoring is based on the first 17 of them (depressed mood, feelings of guilt, suicide, insomnia-early, insomnia-middle, insomnia-late, work and activities, retardation, agitation, anxiety-psychics, anxiety-somatic, somatic symptoms-gastrointestinal, somatic symptoms-general, genital symptoms, hypochondria, loss of weight, and insight). Each item on the questionnaire is scored on a 3- or 5-point scale, depending on the item, and the total score is compared to the corresponding descriptor: 0-7 = normal; 8-13 = mild depression; 14-18 = moderate depression; 19-22 = severe depression; 23-50 = very severe depression.

The Q-LES-Q-SF [43] is a self-report measure of individual's perceived general physical and mental health; it is a 16-item self-administered questionnaire that assesses life satisfaction over the past week. Each question is rated on a 5-point scale from 1 (very poor) to 5 (very good). Scores from the individual items are added together and reported as percentage of the maximum possible score (70 points =100%). The short-form of the Q-LES-Q-SF is identical to the General Activities subscale of the larger Quality of Life Enjoyment and Satisfaction Questionnaire (Q-LES-Q) instrument.

These instruments were administered to the patient at different times: at the moment of hospitalization, at discharge (when the oncologist agreed with Tamoxifen 15, 30, 60, 100, and 365 days after the discharge). Details are reported in Table 1.

## 3. Discussion

We presented the case a 45-year-old male with a diagnosis of BD comorbid with PD, who required repeated hospitalization because of a severe depressive state with mixed features and recurrent suicidal ideation that occurred while the patient was undergoing hormonal therapy with Tamoxifen after surgery for ER+/HER2+ breast cancer. Following the introduction of Tamoxifen to prevent cancer relapse, the patient's depressive and anxious symptomatology was progressively worsening up to the onset of suicidal thoughts. Several therapeutic trials, based on various associations of mood stabilizers, antidepressants, and antipsychotics, did not result in a satisfactory clinical response. The need of two psychiatric hospitalizations in a five-month timespan, for a patient who had previously been followed as outpatient, attests to clinical severity of the clinical picture. XY was firstly discharged with a therapy composed by Divalproex 500 mg/day, Sertraline 50 mg/day (which was later increased), and Olanzapine 7.5 mg/day in addition to Tamoxifen 20 mg/day. Subsequent treatment changes, namely, the addition of Lithium Sulfate to Divalproex as an adjunctive mood stabilizer, the switch from Sertraline to Citalopram, and from Olanzapine to Quetiapine, and the short-term use of benzodiazepines, did not produce any significant improvement. On the contrary, there was a progressive worsening of depressive symptoms with onset of delusions of guilt and suicidal ideation leading to further hospitalization 6 months later. At the moment of the second admission, the psychometric evaluations confirmed XY was suffering from a severe depression; at the same time, he showed a moderate pervasiveness of suicidal thoughts, and the overall Quality of Life was perceived as very poor (see Table 1). Just a mild overall improvement of clinical conditions was observed in response to subsequent therapeutic changes, such as the reintroduction of Sertraline up to 125 mg/day and Olanzapine up to 10 mg/day. At the discharge, the oncologist who was treating the patient approved the discontinuation of Tamoxifen after a multidisciplinary evaluation. While the psychopathological symptoms had been already slightly reducing in response to the therapeutic changes undertaken during hospitalization, after the interruption of Tamoxifen, the scores of MADRS, HAM-D, and Q-LES-Q-SF considerably improved, suggesting a relevant role for Tamoxifen in the and worsening of depressive and anxious symptomatology. This hypothesis is strengthened by the persistence of a stable and satisfactory recovery from the clinical symptoms, along with a further improvement in the perceived quality of life during a 6-month follow-up while the ongoing psychiatric therapy was moderately reduced.

The clinical case we described can be considered to some extent illustrative with regard to the potential risk of developing psychiatric side effects during a long-term intake of Tamoxifen, a risk that, as our case and existing literature show, may be substantially higher in patients with mood disorder. As a matter of fact, XY's case inserts in the patchy literature of case reports describing the occurrence or recurrence of psychiatric symptoms of a depressive or mixed-depressive nature in patients undergoing a Tamoxifen therapy due to oncological indications [23–29]. A further issue deserving attention is the remarkable severity of the clinical picture: similarly to previous case descriptions [23, 25–29, 44–47], the clinical management of our patients resulted to be challenging due to the suicidal ideation and delusional symptoms onset and to the poor therapeutic response.

These findings may be not so surprising if one considers some clinical data and pharmacodynamics aspect. On the one hand, the risk of developing mood episodes in subjects with a bipolar diathesis who were receiving hormonal or immunomodulating treatments for other medical conditions had already been reported (e.g., IFN-*α* treatment for hepatitis C induced major depressive disorder in 12% participants with lifetime subthreshold bipolar disorder symptoms [48]). On the other hand, both the PKC inhibition and the estrogenic modulation are neurobiological mechanisms of major importance, and their effects on mood have been widely reported [33, 49, 50]. In this regard, despite Tamoxifen has been claimed to be a promising antimanic drug, trials were conducted on relatively small samples, and its long-term effects on mood are still largely unexplored [51, 52]; nonetheless, previous studies, yet inconclusive, warned about its potential depressogenic effect [17, 19, 20, 30]. As to the latter issue, further research is warranted in order to obtain a more systematic assessment of Tamoxifen-related depressive risk and to detangle the role of estrogenic deprivation and PKC inhibition in determining it [53–56]. Indeed, literature data focusing on this issue came from researches which are highly heterogeneous as to the assessment method and which usually did not take into account previous psychiatric history [18–20, 22].

Even if it should be taken into account that adverse life events, such a cancer diagnosis, may represent *per se* a trigger for mood symptoms' onset or exacerbation, the temporal relationship with Tamoxifen assumption and the substantial improvement following Tamoxifen discontinuation suggest that it may have a causative role in XY's psychopathological worsening. Furthermore, the gender should be considered as an additional risk factor. This is in line with a previous study by Anelli et al. [30] which reported a lower tolerability of Tamoxifen among male patients: men treated with Tamoxifen seem to develop Tamoxifen-related side effects in a significantly larger proportion with respect to female ones [30], and these side effects are mostly of psychiatric kind (i.e., anxiety and depression) [30, 31].

The scenario we have observed in the clinical management of our patient, combined with the literature, leads us to suggest that patients with BD, especially male ones, deserve a close psychiatric surveillance while undergoing Tamoxifen therapy. Moreover, since the finding of a Tamoxifen-related particularly severe and treatment resistant depression, sometimes complicated by suicidal thoughts, seems not to be an isolated occurrence [57], the possibility of discontinuing or modifying oncologic therapy after a multidisciplinary evaluation should be carefully considered by clinicians. At the same time, these findings stress the need of further studies in order to clarify Tamoxifen's long-term effects in BD and, consequently, to more accurately define the indications of its use in the setting of acute mania.

## Figures and Tables

**Table 1:** Patient MADRS, HAM-D, and Q-LES-Q-SF scores at second hospitalization, discharge, and one-year follow-up.

	MADRS^∗^ range 0-50	HAM-D^∗∗^ range 0-60	Q-LES-Q-SF (% maximum) range 14-70 (0–100%)
Hospitalization	36	28	28 (25%)
Discharge (Tamoxifen interruption)	26	19	34 (47%)
15 days after discharge	15	9	45 (53%)
30 days after discharge	10	3	52 (68%)
60 days after discharge	3	1	63 (88%)
100 days after discharge	3	1	65 (91%)
365 days after discharge	2	1	67 (95%)

^∗^MADRS score: 0-6 = normal; 7-19 = mild depression; 20-34 = moderate depression; 34-60 = severe depression. ^∗∗^Ham-D score: 0-7 = normal; 8-13 = mild depression; 14-18 = moderate depression; 19-22 = severe depression; 23-50 = very severe depression.

## Data Availability

The datasets used and/or analyzed during the current study are available from the corresponding author upon reasonable request.
